# Comparison of the enzymatic efficiency of Liberase TM and tumor dissociation enzyme: effect on the viability of cells digested from fresh and cryopreserved human ovarian cortex

**DOI:** 10.1186/s12958-018-0374-6

**Published:** 2018-06-02

**Authors:** Viola Maria Schmidt, Vladimir Isachenko, Gunter Rappl, Gohar Rahimi, Bettina Hanstein, Bernd Morgenstern, Peter Mallmann, Evgenia Isachenko

**Affiliations:** 1 Research Group for Reproductive Medicine and IVF-Laboratory, Department of Obstetrics and Gynaecology, University Maternal Hospital, Cologne University, Kerpener Str. 34, 50931 Cologne, Germany; 2 Cell Sort Service Department, Center for Molecular Medicine Cologne University (CMMC), Robert Koch Str. 21, 50931 Cologne, Germany

**Keywords:** Artificial ovary, Follicle, Ovarian tissue, Enzymatic isolation, Cryopreservation, Liberase TM, Tumor dissociation enzyme

## Abstract

**Background:**

The aim of this study was to examine the effectiveness of Tumor Dissociation Enzyme (TDE) on the viability of follicles after digestion of fresh and cryopreserved ovarian cortex fragments (OCFs).

**Methods:**

Fresh and thawed OCF from 14 patients (29 ± 6 years), sized 20 to 210 mm^3^ were randomly distributed into four treatment groups and digested with 16% TDE or 0.05 mg/ml Liberase TM: Group 1, frozen OCF digested with TDE; Group 2, frozen OCF digested with LiberaseTM; Group 3, fresh OCF digested with TDE; and Group 4, fresh OCF digested with Liberase TM. Evaluation of follicle viability was performed under light microscope after staining with Neutral red. For visualization of viable and dead cells under a confocal laser scanning microscope, the follicles were stained with Calcein AM and ethidium homodimer-1.

**Results:**

The results showed that the number of retrieved follicles was significantly higher (990 vs 487; *P* < 0.01) in the TDE-treatment group compared to the Liberase TM-group. The presence of intense neutral red stained follicles was significantly higher in Group 1 and Group 3 compared to Group 2 and Group 4 (70.3% ± +/− 6.22 vs 53,1% ± 2.03 and 94.2% ± 6.6 vs 79.1% ± 2.1; *P* < 0.01). The percentage of Calcein AM stained follicles of class V1 was significantly higher in Group 1 and Group 3 compared to Group 2 and Group 4 (95.97% ± 7.8 vs 87.87% ± 2.4; 97.1% ± 6.8 vs 91.3% ± 2.3; *P* < 0.01).

**Conclusion:**

The enzymatic digestion of ovarian cortex with TDE provides recovery of a higher number of healthy preantral follicles in contrast to earlier described Liberase TM procedure.

## Background

Over the last decades, research in medicine has made a great progress in cancer therapy. Long-time survival of cancer patients has increased significantly [1, 2] and after convalescence many patients can lead normal lives. However, highly aggressive and gonadotoxic chemotherapy and radiotherapy have strong side effects such as premature ovarian failure and infertility [3, 4]. At present, the only possibility to maintain fertility as well as hormonal cyclicity and endocrinological function is the cryopreservation and autologous re-transplantation of ovarian cortical fragments taken before chemotherapy or radiotherapy. This method has resulted in large number of live births worldwide [5, 6].

However, this procedure runs the risk of re-transplanting of malignant cells, which makes it unsuitable for patients suffering from neoplastic diseases whose malignant cells are likely to metastasize into the ovary. Cancers that are considered to have a high risk for ovarian metastases are leukaemia, lymphoma, carcinoma coli and cervical as well as uterine cancers [7–10]. In the same time, the re-transplantation of isolated primordial follicles could be an alternative to safe fertility restoration in women with premature ovarian failure [11–13]. It is possible because the basal lamina surrounding the follicles separates them from the surrounding ovarian stroma, blood vessels and nerves, which prevents the invasion of metastatic cells [14]. Therefore, primordial follicles can be isolated and used for the generation of a transplantable artificial human ovary [15, 16]. Construction of an artificial ovary could be the optimal way of preserving fertility as well as protecting female cancer patients, against the re-transplantation of malignant cells, independent from type of neoplastic diseases [9].

This possibility was shown by Laronda et al. [17]. It was demonstrated the real possibility for successful re-transplantation of follicles seeded with scaffolds to SCID-mice with subsequent pups born through natural mating. Successful isolation of preantral follicles from cryopreserved tissue is especially important in global practice: a large amount of ovarian tissues have been already conserved from patients with different types of cancers. According to data, preantral follicles can be successfully cryopreserved before or after their isolation without impairing their ability to survive and grow in vitro [18, 19].

The important step of the artificial ovary creation from preantral follicles is the isolation of these follicles from fresh or cryopreserved (frozen and thawed) ovarian tissues.

In fact, ovarian stromal cells play an important role in the process of development of preantral follicles. According to Young and McNeilly [20], the thecal cells around the secondary preantral follicles appear to be recruited from the surrounding stromal tissue layer by factors that are secreted by activated primary follicles. The growth and development of secondary follicles is affected by steroid hormones that are produced by interactions between granulosa and thecal cells [21, 22]. Ovarian medulla is composed by dense fibrotic stroma consisted from spindle-shaped cells (fibroblasts) and follicles of different stages of maturity [23]. Development of follicles, their ovulation and luteinisation are finely controlled by physiologic hormonal milieu. Therefore, stromal cells of adult ovary actively contribute to the synthesis and phase remodelling both extracellular matrix and blood vessels [23]. Ovarian stromal and endothelial cells are essential to ensure graft survival and quality [24].

In fact, two arteries (*arteria ovarica* and the branch of the *arteria uterina*) penetrate the ovarian *hilus* and form network of blood vessels [23]. They are an opening port for malignant cells [24] which could preferentially generate special kind of stroma cells for aberrant proliferation and invasion [24]. In this case the including of stromal cells by creating of an artificial ovary plays important role only for healthy patients, because of the risk of transfer of malignant cells included in ovarian stroma [7, 8]. The fibroblasts and some other cell types (for example, pre-vascular cells and mesenchymal stem cells) potentially include cancer-associated fibroblasts [25].

At present, for isolation of preantral follicles from human and animal ovarian cortex, a number of mechanical, enzymatic and a combination of both methods have been described [26–40].

The human ovarian tissue has a relatively high density and the current mechanical isolation of follicles demonstrates unsatisfactory performance. However, the combination of mechanical isolation technique with enzymatic digestion significantly improved the viability of isolated follicles [36, 41, 42].

Our own experience supports the effectiveness of the use of a commercial enzyme -cocktail “Tumor Dissociation Enzyme Reagent” (TDE, Innovative Diagnostic Systems Dr. Christian Sartori, Hamburg, Germany) for enzymatic digestion of gynaecological solid tumors with good viability and developmental rate of cancer -and non -cancer cells (non-published data).

This especially designed commercial enzyme - cocktail was developed for gentle enzymatic digestion of solid tumors [43–45] and allows to isolate tumor cells without appearance of apoptosis or necrosis. Despite the highly secured composition of TDE due to patent [46], it was discerned that the contents of this drug are various types of highly purified collagenase (personal communication with Dr. Christian Sartori Labor, Hamburg, Germany) that allowed the digested tissue suspensions to be not sticky and not viscous, and easy to handle and thus, can obtain a good number of viable cells.

The aim of our experiments was to study the Tumor Dissociation Enzyme effects on the integrity and viability of cell -complexes (follicles) after digesting of these cells from cryopreserved ovarian cortex.

## Methods

Except where otherwise stated, all chemicals were obtained from Sigma (Sigma-Aldrich Chemie, GmbH, Schnelldorf, Germany).

### Tissue collection, dissection, and distribution into groups

This study was approved by the Ethics Boards of Cologne University (applications 99,184 and 13–147).

Written informed consents were obtained from all study participants aged 18 and over.

Tissues were obtained from 14 patients aged between 22 and 39 (29.1± 5.9) years. According to our approved protocol, 10% of ovarian tissues (ovarian tissue biopsies, OTBs) collected from patients were used for ‘patient-oriented’ research. This refers to research done in order to assess the viability of the tissues for re-transplantation.

The patients with following diseases were indicated: breast cancer (3 patients), Hodgkin lymphoma (2 patients), non-Hodgkin lymphoma (2 patients), acute lymphoblastic leukemia (3 patient), uterine cancer (1 patient), nasopharyngeal cancer (1 patient) and soft tissue sarcoma (2 patient). For our research, the OTBs from three patients were used after surgery without cryopreservation, and OTBs for patient-oriented research from eight patients were cryopreserved long before these experiments.

The medium used for OTBs transport and dissection (the basal medium) was composed by Leibovitz L-15 with 5% Dextran Serum Substitute (Irvine Scientific, Santa Ana, USA). After collection, fresh ovarian tissue fragments were transported at 32 °C to 34 °C to the laboratory within 20 min of surgery.

Using sterile surgical tweezers and no. 22 scalpels, the medullary part of the fragment was removed to achieve ~ 1 mm thickness of the cortical part. A small piece of fragment (~ 1 mm^3^) was fixed in Bouin solution for histological evaluation and served as a fresh control. Depending on the size of ovarian fragment intended for this research, the size of OCFs for patient-oriented research was ranged from 20 to 210 mm^3^ (Table 1). Each OCF from each patient was cut into two equal parts and cooled to 5 °C for 24 h. After this, the cooled OCF was enzymatically digested or frozen (one OCF per cryo-vial) with subsequent storage in liquid nitrogen until thawing and use. Before each enzymatic treatment, the OCF was weighted using analytical balance.

**Table 1:** Total number of follicles isolated from frozen/thawed ovarian tissues after enzymatic digestion with different kinds of enzymes in 14 experiments

Number of patient	Patient age (years)	Treatment of ovarian cortex	Enzymatic digestion (follicles number)		Volume of piece (mm^3^)	Byopsy weight (g)			Follicle density/(mm^3^)
			TDE**	Liberase TM***		Whole byopsy weight	Byopsy for TDE digestion	Byopsy for Liberase TM digestion	
1	26	frozen	10	5	20	0.0192	0.0090	0.0102	8
2	29	frozen	15	7	30	0.0738	0.0378	0.0360	7
3	33	frozen	23	6	45	0.0941	0.0460	0.0482	7
4	22	frozen	43	20	40	0.0973	0.0479	0.0494	12
5	31	frozen	12	9	22	0.0257	0.0125	0.0132	7
6	39	frozen	14	7	36	0.0854	0.0440	0.0414	5
7	23	frozen	55	13	180	0.2805	0.1396	0.1409	5
8	29	frozen	34	6	55	0.1342	0.0665	0.0677	8
9	34	frozen	125	84	165	0.2348	0.116	0.1188	10
10	20	frozen	321	168	144	0.2220	0.116	0.106	24
11	34	frozen	216	115	157	0.2253	0.1114	0.1139	17
12	32	fresh	44	16	200	0.4592	0.2310	0.2286	4
13	34	fresh	30	12	210	0.5775	0.2905	0.2870	2
14	28	fresh	48	19	55	0.1401	0.0691	0.0710	11
Total	–	–	990	487	–	–			
Mean ± SD			70.7 ± 87.7*	34.8 ± 48.7	81.2 ± 71.8	0.18 ± 0.17	0.096 ± 0.1	0.095 ± 0.1	9.1 ± 5.5

*asterisk corresponding the statistical (*P* < 0.01)

**TDE (Dr. Christian Sartori, Labour, Hamburg, Germany) is a commercial Enzyme-cocktail (Patent Nr. WO 2006031867 A2, 2004)

***Liberase TM Research grade belongs to the group of Liberase Research Grade Purified Enzyme Blends with reduced endotoxine levels and are mixtures of highly purified Collagenase I and Collagenase II, and with a medium concentration of Thermolysin

### Histological examination of ovarian tissue cortex

For histological investigation, the OCFs were fixed in Bouin’ solution, imbedded in paraffin wax, serially sectioned at 5 mm, stained with haematoxylin/eosin, and analyzed under a light inverted stereomicroscope Nikon SMZ1270 (Nikon, Düsseldorf, Germany) under 400× magnification. The following types of preantral follicles were evaluated: (1) primordial follicles composed of an oocyte surrounded by a layer of flattened follicular cells, (2) primary and secondary follicles that are similar to primordial follicles, but in which the oocyte is surrounded by one to two layers of cuboidal granulosa cells. Morphology of the follicles was evaluated on the basis of parameters previously described [47]. The number of viable and damaged follicles was counted. To avoid overcounting of the same follicles, only the section with a visible oocyte nucleus was counted. Normality of follicles was evaluated based on the parameters previously described by Paynter et al. [47]. Three types of follicles were distinguished: Type I follicle is spherical with randomly distributed granulosa cells around the oocytes. The cytoplasm is homogenous with slightly granulated nucleus, in the center of which condensed chromatin is detected in the form of dense spherical structure. Type II follicle is spherical; however, granulosa cells do not cover the oocytes regularly. The oocytes can be flat, and condensed chromatin is not detected in the cytoplasm. Type III follicle has partly or fully disrupted cytoplasm and pyknotic nucleus. The cytoplasm of granulosa cells has damages similar to oocytes. Follicles of Type I and Type II were denoted as normal, and those of Type III were denoted as degenerated.

### Tissue cryopreservation (freezing and thawing)

This procedure was performed as published previously [48–58]. In our protocol, we used DMSO and ethylene glycol as cryoprotective cocktail to support a multi-cellular structure of ovarian tissues to protect all types of cells [59]. On the day of freezing, OCFs were placed for 30 min at room temperature in a 20-ml freezing medium composed of basal medium supplemented with 6% dimethyl sulfoxide, 6% ethylene glycol and 0.15-M sucrose. Then pieces were put into a standard 5-ml cryo-vials (Thermo Fisher Scientific, Rochester, NY, USA) previously filled by 4.5 ml freezing medium and frozen in an IceCube 14S freezer (Sy-Lab, Neupurkersdorf, Austria). The slow cooling profile started at − 6 °C, and the samples were then cooled from − 6 °C to − 34 °C at a rate of 0.3 °C/min. At − 34 °C, the cryo-vials were finally plunged into liquid nitrogen and stored until thawing. The freezing protocol for cryopreservation of this ovarian tissue included an auto-seeding step at − 6 °C.

To thaw the samples, cryo-vials were removed from liquid nitrogen and held for 30 s at room temperature; they were then immersed in a 100 °C (boiling) water bath for 60 s. The exposure time in the boiling water was visually controlled by the presence of ice in the medium; as soon as the ice was 2 to 1 mm apex, the cryo-vial was removed from the boiling water, at which point the final temperature of the medium was between 4 °C and 10 °C. Within 5–10 s after thawing, the pieces from the cryo-vials were transferred to a 10 ml thawing solution (basal medium containing 0.5-M sucrose) in a 100 ml specimen container (Sarstedt, Nümbrecht, Germany). For stepwise dilution of cryoprotectants, the container was placed on a shaker and continuously agitated with 200 osc/min for 15 min at room temperature.

Rehydration of the tissue by stepwise rehydration followed. This was also performed using the same, previously published [5, 48–58] ‘dropping’ methodology: slow addition of basal medium to the solution of sucrose with ovarian pieces. For ‘dropping’, we used 50-ml of basal medium in a 50-ml tube (Greiner Bio-One GmbH, Frickenhausen, Germany). The final sucrose concentration was 0.083-M, resulting in almost isotonic conditions [5, 48–58]. The last step involved three washes in a basal medium for 10 min immediately prior to preparation.

### Preparation of fresh and thawed ovarian tissue and isolation of follicles

We compared the influence of the enzyme Liberase TM Research Grade (Thermolysin Medium Concentration, Roche Diagnostics GmbH, Roche Applied Science, Mannheim, Germany) with the Tumor Dissociation Enzyme (TDE) from the DCS ATP-Chemo-sensitivity Assay Kit (DCS Innovative Diagnostik-Systeme, Dr. Christian Sartori Labor, Hamburg, Germany) on the survival of different stages of isolated follicles after enzymatic digestion of fresh and cryopreserved human ovarian cortex.

The TDE (commercially purchased from the Firma Dr. Christian Sartori Labor, Hamburg, Germany) is a commercial enzyme -cocktail for enzymatic digestion of any solid tumors [43–45].

**Fig. 1:**
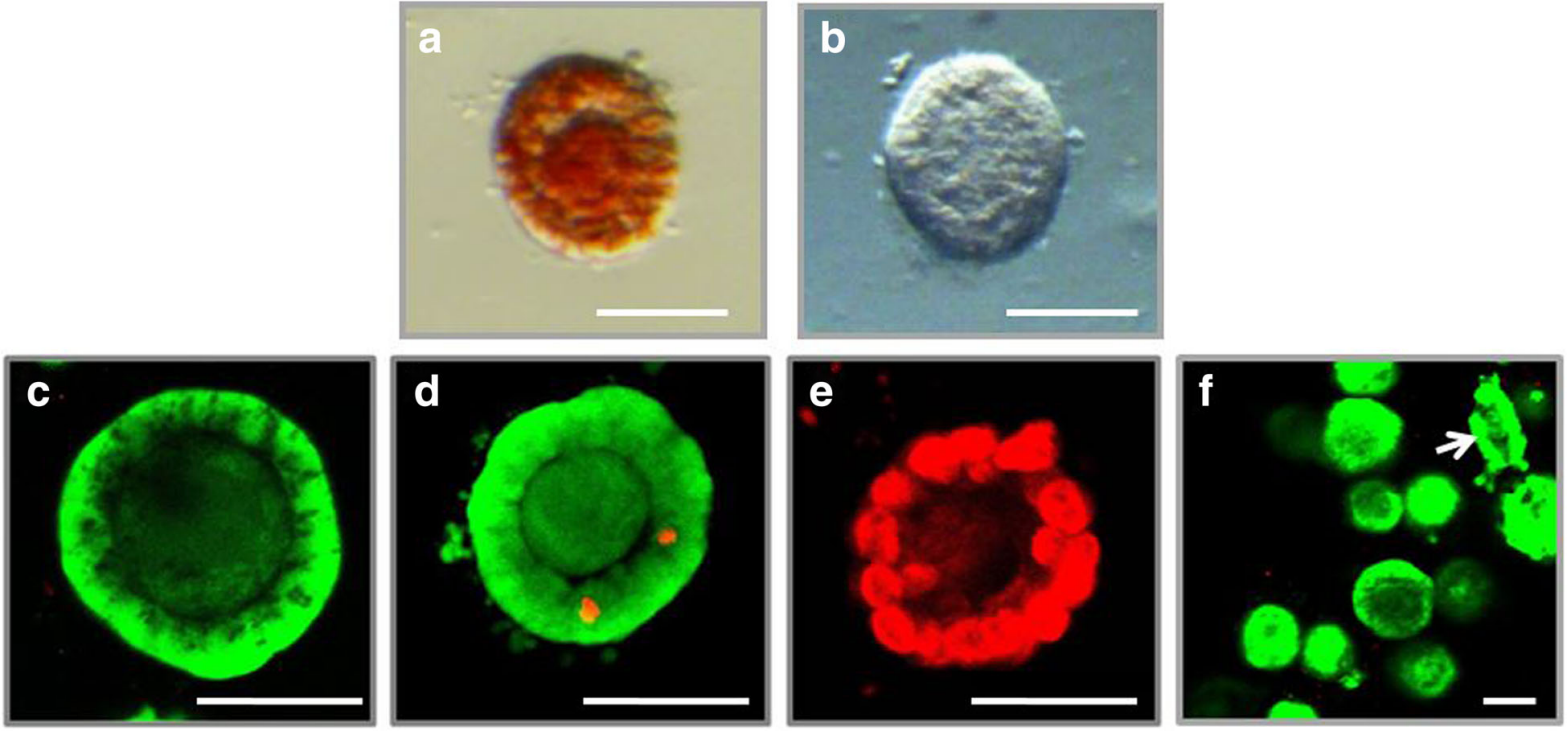
Example of follicles treated with neutral red dye and live/dead fluorochrome assay. **a** neutral red positive follicle; **b** B neutral red negative follicle. Neutral red dye is a viability stain which shows a deep red colour in acid cell structures like lysosomes. **c** V1, fully viable isolated follicle (granulosa cells as well as oocyte show bright green fluorescence) positive to Calcein AM, negative to ethidium homodimer-1; **d** V2, minimally damaged follicle with two cells positive (bright red colour) to ethidium homodimer-1; **e** negative control: dead follicle showing bright red fluorescence (positive to ethidium homodimer-1, negative to Calcein AM); **f** group of follicles, mostly V1, arrow indicates a follicle that was mechanically disrupted during the manipulation process. Bar = 50 μm

All enzyme solutions were prepared on Leibovitz L-15 medium of appropriate concentrations in combination with 50 μg/ml neutral red dye, a vitality dye that shows a deep red colour in acid cell structures for the express- visualization of viable follicles (Fig. 1a, b).

**Fig. 2:**
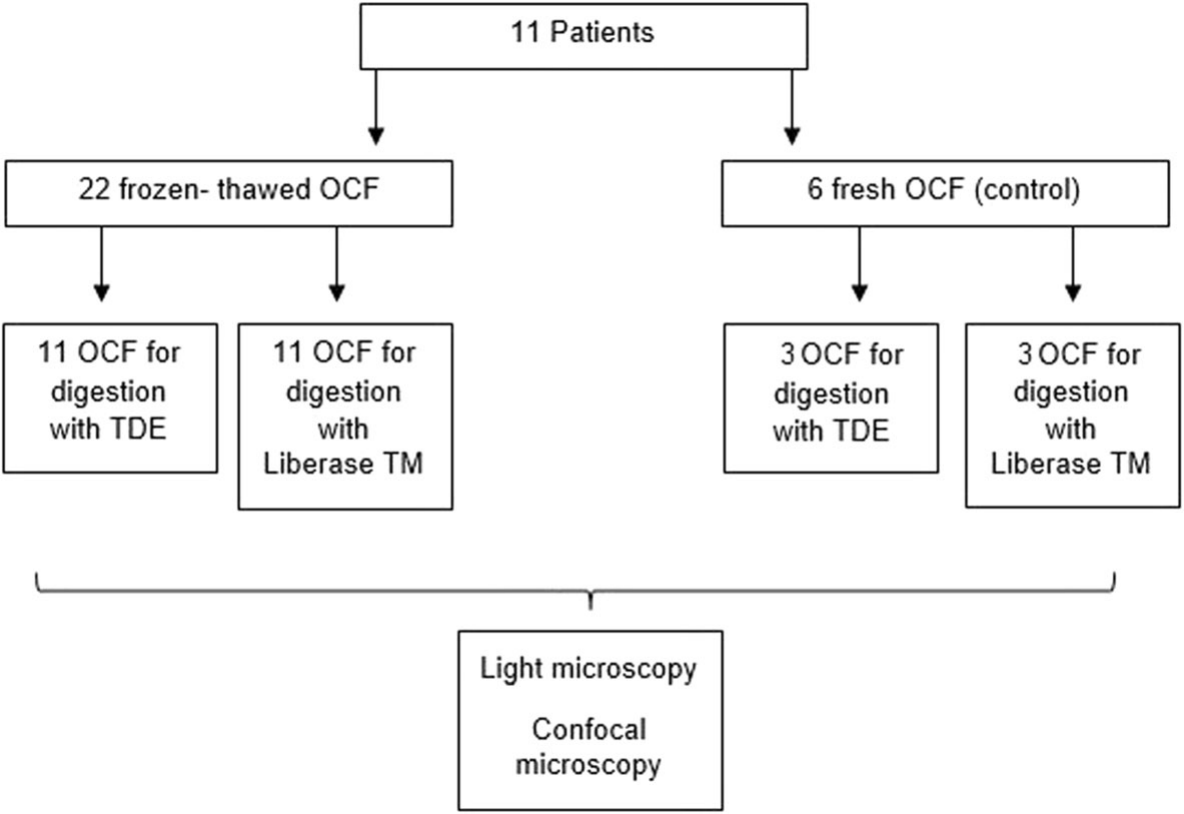
Experimental design

Twenty-two frozen/thawed and six fresh OCFs obtained from 11 patients were divided into four treatment groups: Group 1: 11 frozen OCFs for enzymatic digestion with TDE; Group 2: 11 frozen OCFs for enzymatic digestion with Liberase TM: and two fresh control groups: Group 3: 3 fresh OCFs for enzymatic digestion with TDE; Group 4: 3 fresh OCFs for enzymatic digestion with Liberase TM (Fig. 2).

The treatment procedure was performed as previously described [60] with some modification. OCFs were mechanically cut into ~ 0.5 × 1 to 1 × 1 mm^3^ pieces onto a sterile 100 mm Falcon Petri dish (Falcon, Corning Incorporated-Life Science, Corning, USA) pre-cooled on ice to + 4 °C and put onto a cold plate to stabilize the temperature. The cutting of OCFs was performed with quick simultaneous movements of two scalpels no. 22. The ovarian tissue pieces were then suspended in basal medium containing 16% TDE (Group 1 and Group 3) or in basal medium containing 0.05 mg/ml Liberase TM (Group 2 and Group 4). The optimal concentration for digestion with TDE was titrated in previous experiments (unpublished data). Incubation with enzymes was performed in CO_2_ -incubator at 37 °C on KS 260 basic shaker (IKA, Staufen, Germany) with 100 rotations/min for 75 min. Enzymatic digestion was inhibited by the addition of an equal volume of 4 °C cold Leibovitz L-15 medium containing 20% fetal calf serum (FCS). The cell suspension was immediately put onto ice, gently aspirated up and down with a 1-ml pipette, filtered through 100-μm cell strainers (Falcon, Corning Incorporated-Life Science, NY, USA) and examined by two experienced co-workers under a light inverted stereomicroscope Nikon SMZ1270 (Nikon, Düsseldorf, Germany) in the presence of follicles stained and non-stained with neutral red follicles.

### Collection and morphological evaluation of follicles

Isolated follicles were collected with 135 μm V-denuded capillaries (Vitromed GmbH, Jena, Germany) and washed four times in pre-cooled to 4 °C basal medium to discard stromal cells. The number of stained and unstained follicles was calculated for both groups. To identify the isolated primordial follicles, the classification described by Gougeon and Fortune [61, 62] was used: primordial follicle (< 60 μm), oocytes surrounded by a single layer of flattened pre-granulosa cells; primary follicle (> 60 μm - ≤75 μm), oocytes with single layer of cuboidal granulosa cells; secondary follicle (> 75 μm - < 200 μm).

Immediately after isolation of follicles, they were observed under Nikon microscope SMZ25. The follicle diameter was measured by Program Zen (Nikon, Düsseldorf, Germany).

### Confocal laser scanning microscopy

The neutral red staining method provides a fairly rough estimation of viability. However, dead granulose cells are difficult to identify [39] and we have chosen a live/dead fluorochrome assay for exact analysis of viability using confocal laser scanning microscopy. To check the viability of cellular structures of isolated ovarian follicles (oocytes surrounded by flat or cubical follicular cells) collected follicles were stained with Calcein AM for visualization of viable cells and ethidium homodimer-1 for visualization of dead cells as previously described by Cortvrindt and Smitz [63]. The follicles were exposed to 2 μM of Calcein AM and 5 μM of ethidium homodimer-1 in Dulbecco’s phosphate buffer saline (DPBS) for 15 min at 37 °C in the dark. During this time, living cells for intracellular esterase activity converted the non -fluorescent cell-permeable Calcein AM in fluorescent Calcein producing an intense bright uniform green fluorescence (ex/em, 495 nm/515 nm). In contrast to Calcein AM, ethidium homodimer-1 shows a red fluorescence when bound to the DNA of dead cells (ex/em, 528 nm/617 nm) (Fig. 1c-f). After exposure to these dyes, the follicles were washed in PBS and visualized using a confocal laser scanning microscope Olympus Fluoview FV 1000 (Olympus, Hamburg, Germany) with a multi-line argon laser (458, 488, 515 nm) to record the fluorescent images. Follicle viability was done as described by Paulini [64], as follows. Viable follicles of class V1: oocytes and all granulose cells are viable; minimally damaged follicles of class V2: the presence of less than 10% dead follicular cells; moderately damaged follicles of class V3: 10–50% dead follicular cells; and follicles of class V4: both the oocytes and all follicular cells are dead.

Confocal images were processed and analyzed using the program Fiji, an open-source platform for scientific image processing, which is an advanced version of the commonly used Image J.

### Statistical analysis

The enzymatic digestion of ovarian cortex with two types of enzymes was repeated at least three times on different days. Results were expressed as mean ± standard deviation (SD). Statistical evaluation of flow cytometrical results was performed with the GraphPad Prism 5 software package (GraphPad, La Jolla, USA), applying D’Agostino’s K2 test to assess Gaussian distribution. To evaluate the effect of two different enzymatic treatments on the cellular viability of ovarian cortex (stromal cells and follicles) immediately after digestion, a T-test for equal variances was performed. *P*-values less than 0.05 were considered statistically significant.

The correlation test (EXCEL – 2010) was performed for multi-variables (recovered follicle number, patient’s age, size and volume of biopsies) to investigate the relationship between these parameters. The value of correlation coefficient (r) was always between + 1 and − 1 inclusive, where 1 is total positive linear correlation, 0 is no linear correlation, and − 1 is total negative linear correlation. A commonly used scale for the interpretation of correlation coefficient is the following: no correlation: r from 0 to 0.1 or from 0 to − 0.1; weak correlation exists: r from 0.1 to 0.3 or from − 0.1 to − 0.3; moderate correlation exists: r from 0.3 to 0.5 or from − 0.3 to − 0.5; and strong correlation exists: r from 0.5 to 1 or from − 0.5 to − 1.

## Results

### Retrieval rate of isolated follicles

In experiments total of 1477 follicles were isolated from fresh and frozen OCFs (169 follicles were isolated from 6 fresh and 1308 follicles were isolated from 22 cryopreserved OCFs). From fresh biopsies, 122 follicles were recovered using TDE and 46 follicles were recovered using Liberase TM. From frozen biopsies, 868 follicles were recovered using TDE and 440 follicles using Liberase TM (*P* < 0.05) (Table 1). It was established that the retrieval rate of follicles from the biopsies of patients 22–39 years old has a strong negative (*r* = − 0.6) correlation with age. However, the relationship between the patient age and number of follicles per 1 mm^3^ had no correlation (*r* = 0.06). The correlation between the volume of biopsies and number of retrieved follicles is a moderate positive (*r* = 0.3). The same moderate correlation (*r* = 0.4) was found between the volume of biopsies and number of follicles per 1 mm^3^. The correlation between the weight of biopsies and number of retrieved follicles was very weak (*r* = 0.1). However, between the weight of biopsies and number of follicles per 1 mm^3^, medium correlation (*r* = 0.) was found. Between the age of patient and volume of biopsies or weight of biopsies, the same very weak correlation (*r* = 0.1 and *r* = 0.2, respectively) was observed. However, the relationship between the weight of biopsies and its volume has a strong positive correlation (*r* = 1) (Table 1).

### Quantity and morphology of isolated follicles

**Fig. 3:**
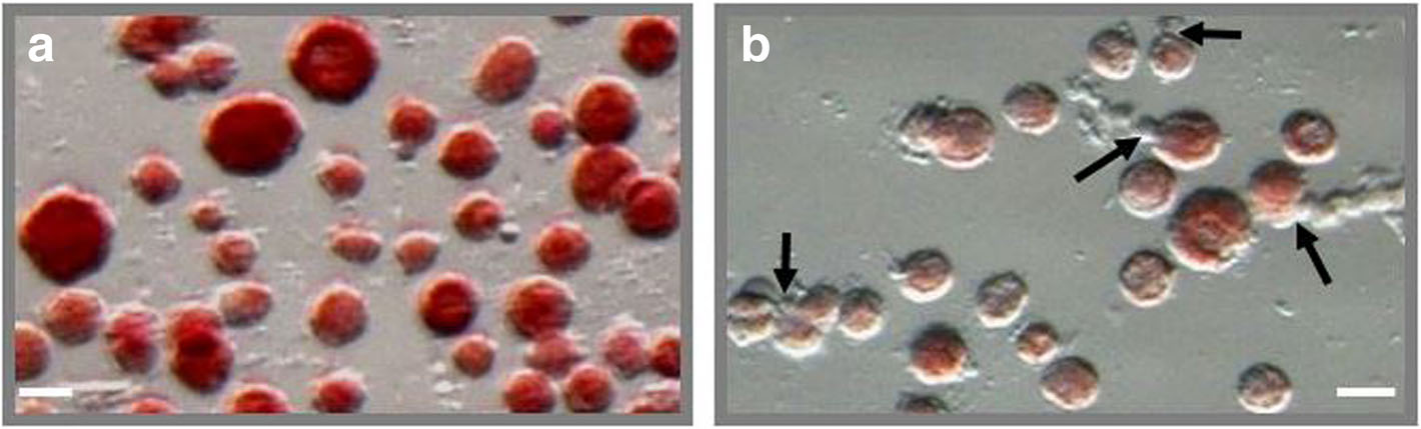
Typical view of follicle suspension after enzymatic digestion. **a** follicles isolated from frozen ovarian cortex with TDE-enzyme cocktail; **b** follicles isolated from frozen ovarian cortex with Liberase TM. The black arrows show the clustered and partially isolated from incompletely digested stroma follicles. Bar = 50 μm

As shown in Fig. 3a the most of follicles in Group 1 are fully isolated (*P* < 0.01).

**Fig. 4:**
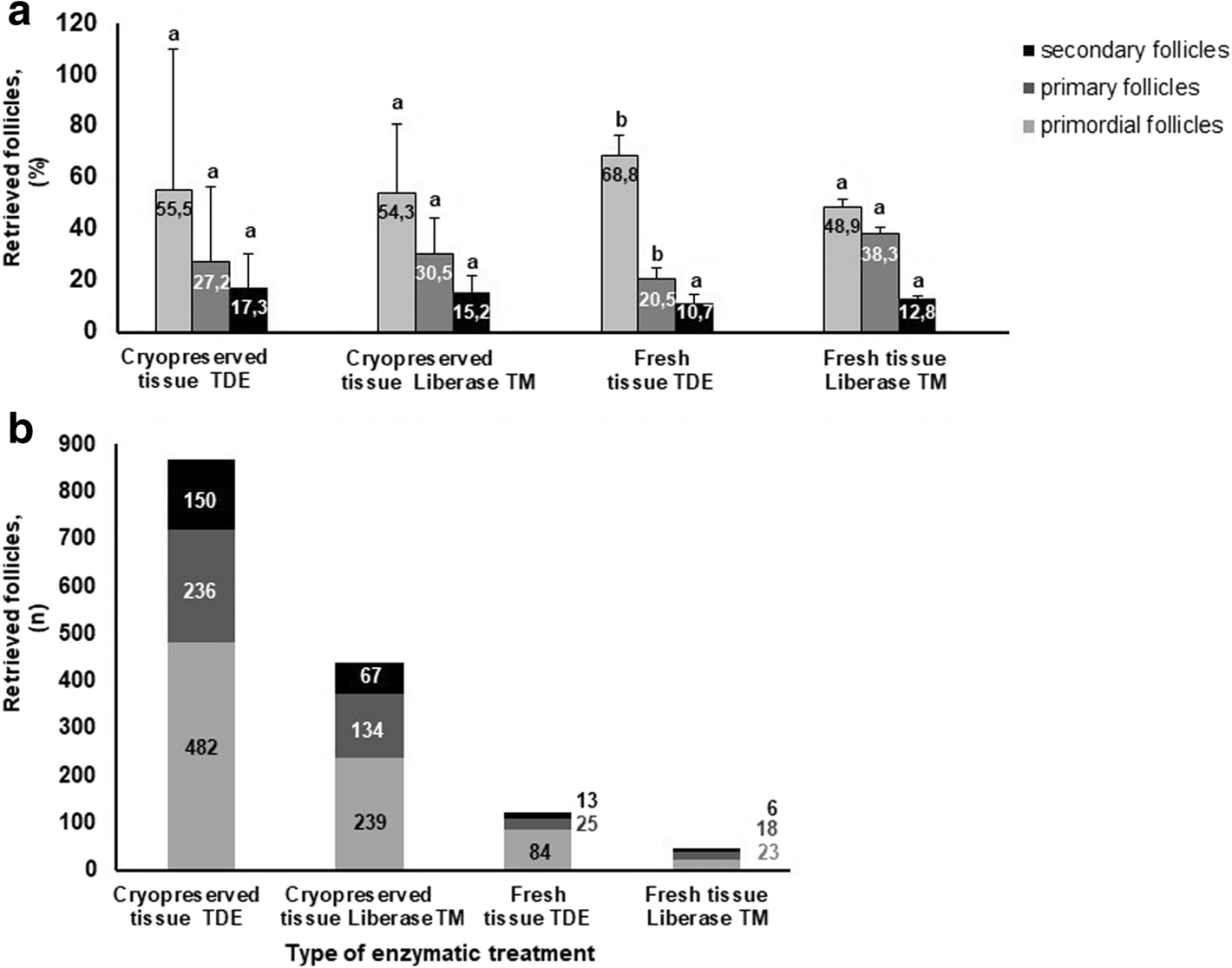
Distribution of follicles according to their maturity in each treatment group. **a** Percent of retrieved follicles of different maturity inside of each treatment group. **b** Number of retrieved follicles of different maturity inside of each treatment group. Bars (mean ± SD) with different superscripts in respective treatment group represent significant differences (*P* < 0.05)

Compared to digestion with TDE, the digestion with Liberase TM (Fig. 3b) has resulted in incomplete tissue digestion (black arrows). Extruded oocytes were found in both treatment groups, but it was extremely rare (< 3%). Note that apart from the good three-dimensional structure, the general morphology of isolated follicles was well maintained independently from the type of enzymatic treatment. After evaluation of isolated follicles under an inverted microscope, it was noted their normal spherical form with mostly one layer of granulosa cells around the oocyte. The data for the quantity of retrieved follicles of different maturity inside each treatment are shown (Fig. 4a). It was also demonstrated that the number of retrieved follicles independently from their maturity was significantly (*P* < 0.01) higher in the TDE -treatment group in comparison with Liberase TM groups (Group 1[*n* = 122] vs Group 2 [*n* = 46], Group 3 [*n* = 868] vs Group 4 [*n* = 440]. However, these differences in all treatment groups were no significant (*P* > 0.1) in the distribution of follicles according to their maturity (Fig. 4b).

### Viability of follicles

The viability assessment of follicles was performed using two techniques: (1) express technique for visualization of live follicles with the application of neutral red dye and subsequent evaluation of follicles under a light inverted stereomicroscope and (2) by fluorescence-staining technique with Calcein AM and ethidium homodimer-1 for visualization of viable and dead cells under a confocal laser microscope.

### Express technique for visualization of follicle vitality

Immediately after enzymatic treatment and simultaneous staining with Neutral red dye, the suspension of ovarian stromal cells and follicles were examined under an inverted microscope. Follicles were collected and neutral red -uptake was investigated based on the optical presence of their red staining.

**Fig. 5:**
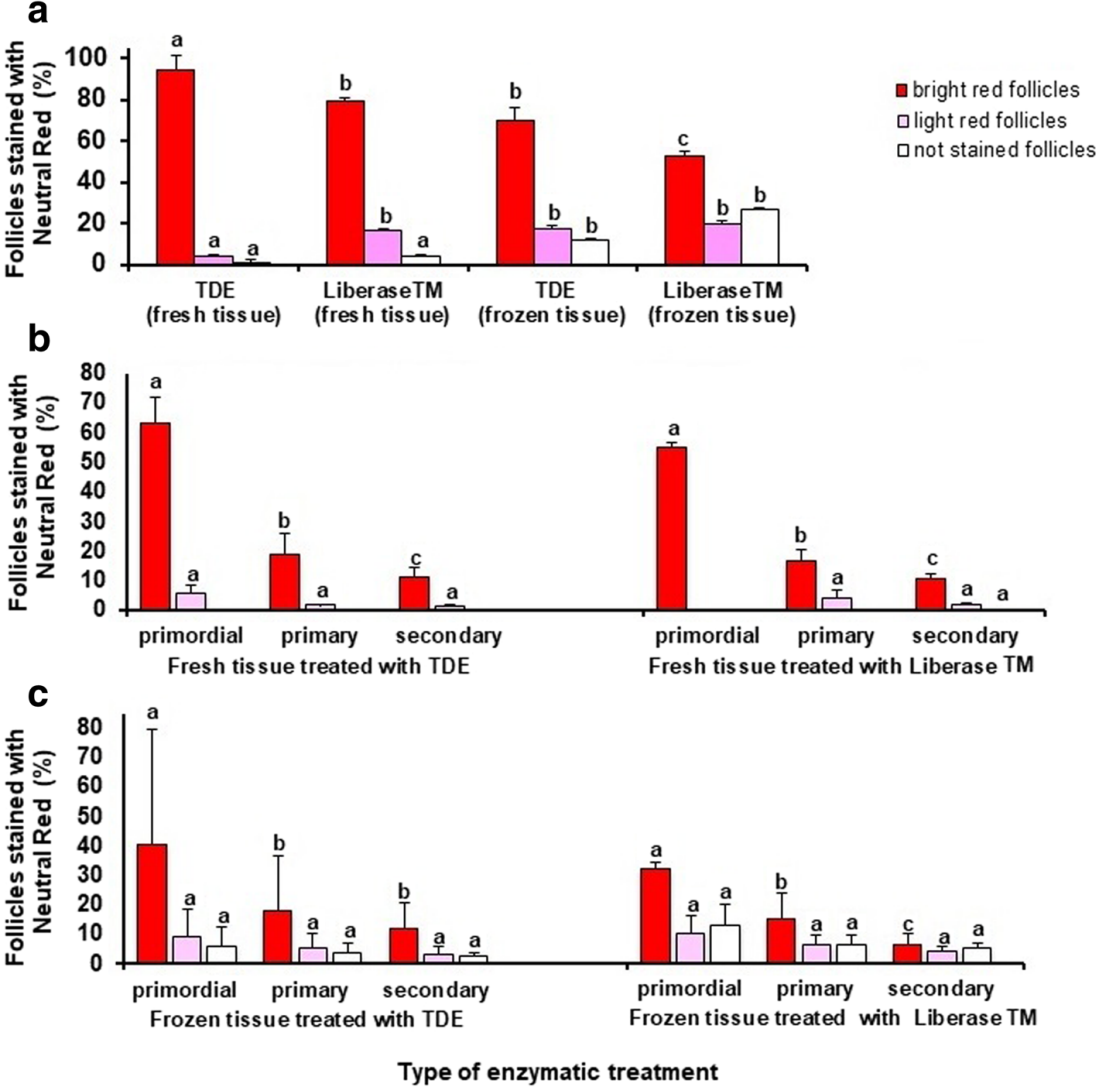
Influence of the type of enzymatic treatment of ovarian cortex on the vitality of isolated follicles of different maturity tested applying of Neutral Red dye. **a** Vitality of follicles in different treatment groups independent of their maturity stage; **b** Comparison of follicle vitality in Group 1 (fresh ovarian tissues digested with TDE) compared to Group 2 (fresh ovarian tissues digested with Liberase TM) depending on their maturity stage, **c** Comparison of follicle vitality in Group 3 (frozen ovarian tissues digested with TDE) compared to Group 4 (frozen ovarian tissues digested with Liberase TM) depending on their maturity stage. Bars (mean ± SD) with different superscripts in respective treatment group represent significant differences (*P* < 0.05)

The data on Fig. 5a show that the presence of intense red -stained follicles in the suspension from fresh ovarian tissues digested with TDE was significantly higher than in the suspension of tissues digested with Liberase TM (94.2 ± 6.6% in Group 1 vs 79.1 ± 2.1% in Group 2, respectively).

The percent of light red- stained follicles was significantly higher (*P* < 0.05) in the suspension of tissues digested with Liberase TM than after digestion with TDE (Group 2 vs Group 1: 16.6 ± 0.6% vs 4.3 ± 0.4%, respectively), while the presence of non -stained follicles was not significantly different in these groups (Group 2 vs Group 1: 4.2 ± 0.3% vs 1.4 ± 0.3%, respectively). The presence of intense red -stained follicles in the suspension of cryopreserved ovarian tissues digested with TDE was significantly higher (*P* < 0.05) in frozen ovarian tissues (70.3 ± 6.2% in Group 3 vs 53.1 ± 2.0% in Group 4, respectively) than in the suspension of tissues digested with Liberase TM.

The amount of light red -stained follicles was not significantly different (*P* > 0.1) between Group 3 and Group 4 (17.7 ± 1.5% vs 20.2 ± 1.0%, respectively), while the presence of non-stained follicles was significantly higher (*P* < 0.05) (12.0 ± 1.3% in Group 3 vs 26.8 ± 0.9% in Group 4, respectively) in the suspension of tissues digested with Liberase TM. The data on Fig. 5b (digested fresh tissue) and Fig. 5c (digested frozen tissue) characterize the vitality of follicles according to their maturity. No significant difference (*P* > 0.1) according to the vitality of different maturity stages of follicles between treatment groups was found.

### Vitality visualisation with confocal laser scanning microscopy

**Fig. 6:**
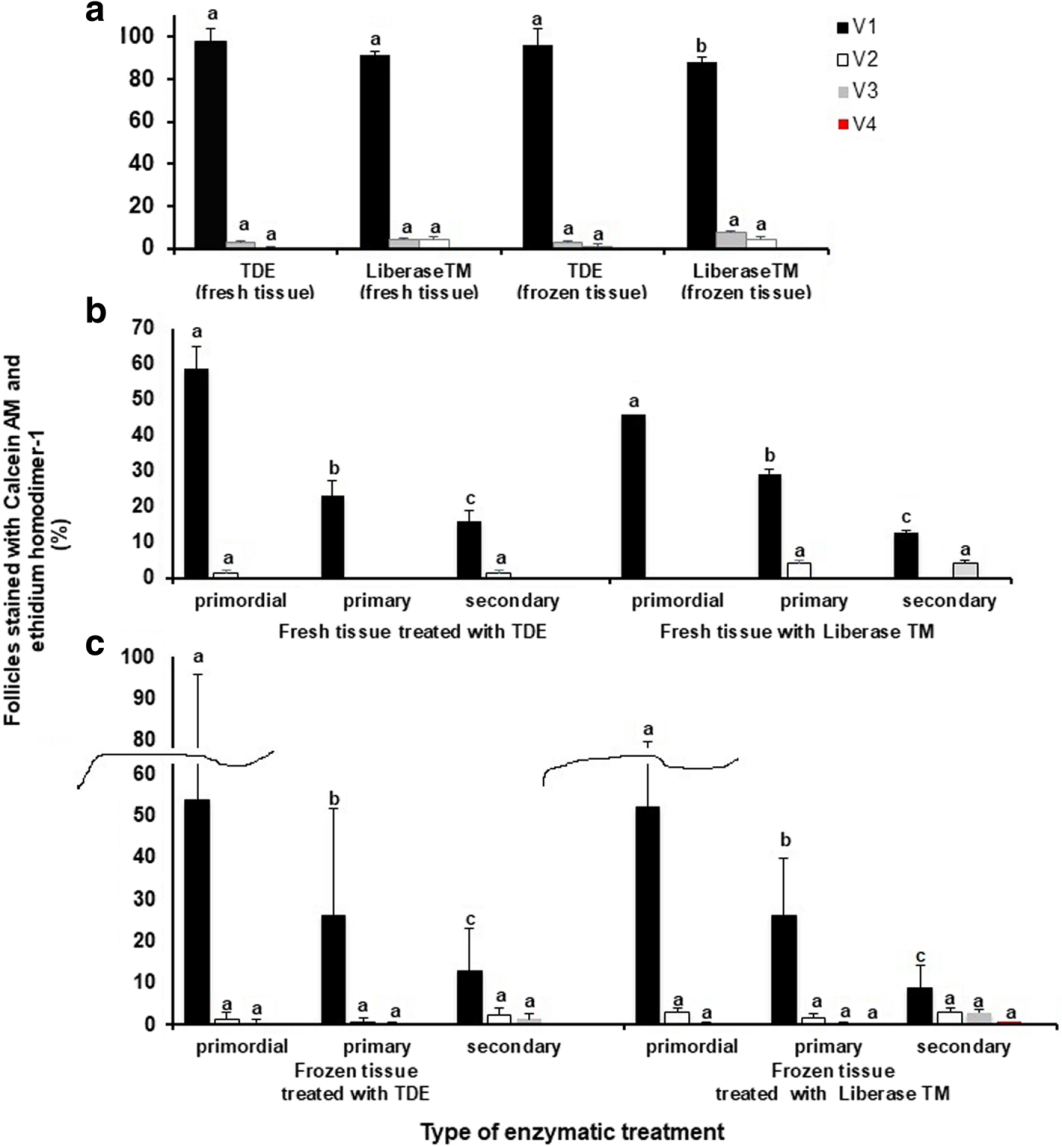
Influence of the type of enzymatic treatment of ovarian cortex on the vitality of isolated follicles of different maturity tested using of Calcein AM for visualization of viable cells and ethidium homodimer-1 for visualization of dead cells. **a** Viability of follicles in different treatment groups. **b** Comparison of follicle vitality in Group 1 (fresh ovarian tissues digested with TDE) compared to Group 2 (fresh ovarian tissues digested with Liberase TM) depending on their maturity stage, **c** Comparison of follicle vitality in Group 3 (frozen ovarian tissues digested with TDE) compared to Group 4 (frozen ovarian tissues digested with Liberase TM) depending on their maturity stage. Bars (mean ± SD) with different superscripts in respective treatment group represent significant differences (*P* < 0.05)

All isolated follicles after assessment of vitality using the Neutral red dye were stained with fluorescent dye to identify the presence of living and dead cells in each follicle. The data on Fig. 6a show that the percent of recovered class V1 follicles in the suspension from fresh ovarian tissues are not significantly different between Group 1 and Group 2 (97.1 ± 6.8% vs 91.3 ± 2.1%, respectively) (*P* > 0.1). However, a significantly higher rate (96.0 ± 7.8% vs 87.9 ± 2.4%, respectively) (*P* < 0.05) of recovered follicles of class V1 in Group 3 (cortical tissue suspension digested with TDE) compared to Group 4 was found. The amount of class V2 and V3 follicles in all treatment groups was not significantly different (*P* > 0.1). Class V4 follicles were absent in all treatment groups.

The data on Fig. 6b (digested fresh tissue) and Fig. 6c (digested frozen tissue) characterize the vitality of follicles according to their maturity stage.

No significant difference (*P* > 0.1) between class V1 primordial follicles was found in ovarian tissues of Group 1 (fresh, TDE-digested ovarian tissues) (58.5 ± 4.2%), Group 2 (fresh, Liberase TM-digested ovarian tissues) (55.8 ± 1.3%), Group 3 (frozen, TDE-digested ovarian tissues) (53.9 ± 4.2%), and Group 4 (frozen, Liberase TM-digested ovarian tissues) (52.1 ± 1.3%).

The presence of class V2 and V3 follicles was not significantly different between all treatment groups, independent of the stage of preantral follicles, (*P* > 0.1). Class V4 preantral follicles in all treatment groups were absent.

### Histological evaluation of fresh and frozen ovarian tissue

Histological evaluation of non-treated pieces of ovarian cortex (fresh control) shows that the most of exanimated follicles (96.8 ± 2.5%) were morphologically normal.

Histologic analysis of haematoxylin-eosin stained ovarian cortical tissues showed morphologically normal preantral follicles. The follicles were surrounded by non-disrupted intact basement membrane. The oocytes were slightly stained, without signs of degeneration or retraction. The percentage of morphologically normal follicles was not significantly different (*P* > 0.1) between the fresh and frozen ovarian tissues samples (96.8 ± 2.5% vs 97.1 ± 5.1%, respectively).

## Discussion

The latest work in the field of follicle isolation from human and animal ovarian cortical tissue indicates their excellent survival after thawing and the ability to further development both in culture and after xenotransplantation [18, 19, 64, 65]. An important task is isolation of the follicles from ovarian cortex to recover a maximal number of high quality viable follicles.

In this work, we applied our original routine cryopreservation protocol for human ovarian tissue, which presupposes the long-time tissue pre-cooling step before freezing. The reason is our previous establishment that the 24 h cooling to 5 °C before cryopreservation is beneficial for the cryopreservation of human ovarian tissues, especially of follicles [5, 48–58].

Enzymatic digestion is commonly used for tissue dissociation and cell harvesting and offers the advantages of unattended quick sample preparation, potential automation, and is low cost with the possibility to obtain more cells of interest [66–68]. The feasibility of enzymatic digestion is a good alternative tissue sample preparation method to the technique of mechanical tissue dissection.

It is well known that the human ovarian cortex possesses a highly dense and fibrous structure. Therefore, using of special enzymatic digestion technique is necessary for effective isolation of the follicles from the surrounding tissue.

The widely used enzyme collagenase is known to degrade connective tissues to allow tissue dissolution and to get the single-celled suspensions. However, enzymatic digestion with the use of collagenase also has serious disadvantages. These disadvantages are well described [69] on the example of hepatocytes.

Thus, it is known that most isolation protocols result in damage of cell junctions, cell membranes, to cell surface receptors and antigens, and cytosolic contents [70, 71].

By enzymatic follicle isolation, the basal membrane disruption occurs often [72]. Collagenase digestion also induces oxidative stress observed 4-8 h after isolation of hepatocytes, leading to a loss of cytochrome enzyme activity [73].

This could be why many isolated preantral follicles degenerate within the first 24 h of in vitro culture and only a few of them could reach the early antral stage [34, 35, 38, 41].

In attempts to standardize the protocol of enzymatic digestion and to improve the quality of isolated follicles, the various types of collagenase (Ia, II, IX, XI) alone [36, 37, 39, 74–76] or in combinations with DNA-se [35, 38, 41, 76–78] were used. However, it was reported about increased amount of lipid droplets in granulosa cells of isolated follicles (collagenase IX and deoxyribonuclease IV, [35]) and about high amounts of premature oocyte extrusions from the enzymatically isolated follicles (collagenase Type II) [37] that shows that the enzyme collagenase has a batch-to-batch variation in effectiveness [79].

According to the latest data [36, 40, 42, 80], the viability of enzymatically isolated follicles depends on the level of purity, the type of collagenase and on combination with other enzymes, which could reduce its toxicity. Such products are Liberase Research Grade Purified Enzyme Blends and are mixtures of highly purified collagenase and neutral protease enzymes, formulated for efficient, gentle, and reproducible dissociation of tissues from a wide variety of sources (Roche Diagnostics GmbH, Mannheim, Germany).

The works of Dolmans et al. [36], Vanacker [40] and Kristensen [39] have shown the beneficial role of different types of Liberase for follicle isolation during enzymatic digestion. In the work of Lierman [60] both Liberase TM combined with collagenase IV and Liberase DH were shown to be better for isolating high-quality primordial follicles, compared with mono-enzyme collagenase IV. The Liberase TM (Thermolysin Medium, 0.04 mg/ml, Roche Diagnostics GmbH, Mannheim, Germany), we used in our experiments belongs to the group of Liberase Research Grade Purified Enzyme Blends with reduced endotoxin levels and is a mixture of highly purified Collagenase I and Collagenase II with a medium concentration of Thermolysin (a non-clostridial neutral extremely stable Zn-metalloendopeptidase (www.Sigma-Aldrich.de) formulated for efficient, gentle, and reproducible dissociation of tissues from a wide variety of sources (http://www.roche-applied-science.com; Worthington Enzyme Manual, V Worthington Biochemical Corporation). Their activity is directed to break the peptide bonds in collagen (Collagenase), fibronectin, collagen IV, and to a lower extent collagen I, however, it does not cleave collagen V or laminin of neutral protease (V Worthington Biochemical Corporation).

Compared to Liberase TM, TDE is a commercial enzyme -cocktail [46] for enzymatic digestion of any solid tumors [43–45]. Due to highly secured patenting [46], we could not obtain the full composition of this enzyme cocktail and we can only assume, according to the quality of the digested material (not sticky, not viscous, and easy to handle), that this cocktail, together with highly purified types of collagenase, may also include enzymes such as protease, dispase or DNA-se.

The comparison of two digestion protocols according to the quality of digestion of stromal tissues showed that TDE allows complete digestion of the stromal tissue with good preservation of follicle integrity. The digested tissue suspension is easy to handle, not sticky and able to obtain a good number of viable follicles. In contrast, the tissue digested with Liberase TM remained poorly dissolved, was sticky and stretchy, and many follicles were still tightly embedded in the tissue and was very difficult to isolate, and this support the finding described [39]. The comparison of the two treatment groups also showed that the number of fully isolated follicles in the TDE groups was significantly higher than in the Liberase TM groups both for fresh and frozen tissues, considering that the size of digested ovarian cortex was similar in both groups.

For evaluation of the quality of recovered follicles from cryopreserved ovarian cortex, we used two techniques: (1) vital staining with neutral red dye for visualization of live follicles and (2) fluorescence -staining of follicles with Calcein AM and ethidium homodimer-1 for visualization of viable and dead cells under a confocal laser microscope.

We have decided to apply the neutral red dye in our experiments, because this vital dye has no deleterious effects on enzymatic activity within cellular organelles [81] and proves to be nontoxic with no long-term negative effects on the follicles [39, 82]. Neutral red (toluylene red, Basic Red 5, or C.I. 50,040) is a eurhodin dye used for many staining methods in histology. Neutral red is used also as a vital stain because the live cells incorporate neutral red into their lysosomes [83]. As cells begin to die, their ability to incorporate neutral red diminishes [84, 85]. This vital dye allows also easy visualization of the follicles in cell suspension. This technique was first applied by Kristensen [39] for follicle visualization and their vitality assessment, and it allows accelerating the process of follicle collection. Our results show that the presence of intense red stained follicles in the ovarian cortical tissue suspension digested with TDE for both fresh and frozen ovarian tissues was significantly higher than in the suspension of tissues digested with Liberase TM.

Visualization of viable and dead cells under a confocal laser scanning microscopy with Calcein AM and ethidium homodimer-1 showed that digestion ovarian tissue with TDE for both fresh and frozen samples provides a significantly higher integrity rate of recovered follicles (class V1) compared to the Liberase TM -groups. The effectiveness of the TDE technique is similar to the data for the Liberase follicles isolated from fresh cortical tissues [36] and with the data for follicles isolated from frozen tissues [18, 86–88].

According to the latest research data, the best survival of the cells of an oocyte-granulosa-cells complex immediately after enzymatic isolation of preantral follicles from fresh ovarian tissue with subsequent one-week in vitro culture provides the Liberase DH enzymatic cocktail [40]. Our data presented here with use of TDE commercial enzyme-cocktail for isolation of preantral follicles are not principally different from the results achieved with use of Liberase DH for digestion of fresh ovarian tissue (95% TDE vs 95% Liberase DH, *P* > 0.5) [40]. The use of highly purified commercial enzyme-cocktails allows eliminating the possibility of obtaining undesirable results through the fault of the drug manufacturer [35, 37, 41, 79]. In fact, there are not principal differences between these two enzymatic cocktails: they content seemingly different types of collagenase.

In the future work we suppose to perform a comparative evaluation of effectiveness of these two commercial enzymatic cocktails (TDE and Liberase DH) with detailed investigation of all apoptotic degenerations (early apoptotic, late apoptotic and necrotic cells) which could take place in enzymatically isolated follicles using specific surface markers for follicular cells by applying flow cytometry and confocal microscopy.

## Conclusion

New method for isolation of preantral follicles with tumor dissociation enzyme (TDE) that can be used for the construction of artificial ovary, provides a higher number of healthy preantral follicles from cryopreserved ovarian cortex in contrast with the earlier described Liberase Thermolysin Medium method.

## Abbreviations

DNA: Deoxyribonucleic acid
Liberase TM: Liberase Thermolysin Medium
OCF: Ovarian tissue cortical fragment
OTB: 24 ovarian tissue biopsies
PBS: Phosphate buffer saline Dulbecco
SD: Standard deviation
TDE: Tumor dissociation enzyme
